# Germline TP53 p.R337H and XAF1 p.E134* Variants: Prevalence in Paraguay and Comparison with Rates in Brazilian State of Paraná and Previous Findings at the Paraguayan–Brazilian Border

**DOI:** 10.3390/curroncol32060333

**Published:** 2025-06-06

**Authors:** Edith Falcon-de Legal, Marta Ascurra, Rosa Vega-Paredes, Elis Sade, Magna Monteiro, Mariana Paraízo, Magali Colman, Angeles Gutierrez Florentin, Cesar Ojeda, Horacio Legal-Ayala, Andreas Ries

**Affiliations:** 1 Laboratorio de Bio y Materiales, NIDTEC-Núcleo de Investigación y Desarrollo Tecnológico, Facultad Politécnica, Universidad Nacional de Asunción, Campus UNA, San Lorenzo 111421, Paraguay; mmonteiro@pol.una.py; 2 Programa de Prevención de la Fibrosis Quística y del Retardo Mental, Centro de Salud No. 9, Ministerio de Salud Pública y Bienestar Social, Asunción 001222, Paraguay; marta.ascurra@gmail.com; 3 Departamento de Bioquímica Clínica, Universidad del Norte, Asunción 001218, Paraguay; rosamariavega14@gmail.com; 4 Hospital de Clinicas, Universidade Federal do Paraná, Curitiba 80060-000, Brazil; elisrosane@hotmail.com; 5 Instituto de Pesquisa Pelé Pequeno Príncipe, Curitiba 80250-060, Brazil; mmparaizo@gmail.com; 6 CEBIOTEC-Centro de Investigación en Biotecnología e Informática, Asunción 001226, Paraguay; magicolman092@gmail.com (M.C.); olangie12@gmail.com (A.G.F.); prof.ceoj93@gmail.com (C.O.); 7 NIDTEC-Núcleo de Investigación y Desarrollo Tecnológico, Facultad Politécnica, Universidad Nacional de Asunción, Campus UNA, San Lorenzo 111421, Paraguay; hlegal@pol.una.py (H.L.-A.); andreas.ries@pol.una.py (A.R.)

**Keywords:** TP53 p.R337H variant, XAF1 p.E134* variant, cancer, Li–Fraumeni syndrome, adrenal gland

## Abstract

Adrenal cortex carcinoma (ACC) in children is a rare tumor that is probably of multifactorial origin and is mainly associated with genetic and environmental alterations. In the south and part of the southeast of Brazil, as well as in the Paraguayan region bordering the Brazilian State of Paraná, ACC prevalence is higher than in any other country, which is associated with the high prevalence of the TP53 p.R337H variant in Paraná (0.30%), Santa Catarina (0.249%), cities around Campinas-SP (0.21%), and the Paraguayan cities on the border with Paraná (0.05%). Recent research suggests that the co-segregation of XAF1-E134* and TP53-R337H mutations leads to a more aggressive cancer phenotype than TP53-R337H alone. Breast cancer may be mildly influenced by co-segregation with XAF1 p.E134*, and this variant can also confer risk for sarcoma. Objectives: The objectives of this study were to (1) estimate the prevalence of the germline TP53 p.R337H and XAF1 p.E134* variants in Paraguay (excluding cities on the border with Paraná State, Brazil) and (2) estimate whether the ethnic origin of TP53 p.R337H carriers in Paraguay is similar to that of ethnic groups in Paraná (possible Portuguese/Spanish origin). Materials and methods: DNA tests for the identification of TP53 p.R337H were carried out from 2016 to 2019 at the Bio-Materials Laboratory of Facultad Politecnica, UNA, and at the Research Center in Biotechnology and Informatics (CEBIOTEC), Asunción, Paraguay. Polymerase chain reaction followed by restriction enzyme digestion (PCR-RFLP) was used to identify TP53 p.R337H, and real-time PCR (RT-PCR) was employed for XAF1 p.E134*. Peripheral blood samples from 40,000 Paraguayan newborns (NBs) were used for the TP53 p.R337H tests. The XAF1 p.E134* tests (RT-PCR) were performed on samples from 2000 Paraguayan newborns at the Pelé Pequeno Principe Research Institute, Curitiba, PR, Brazil. Results: The TP53 p.R337H variant was not found in any of the 14 Paraguayan departments investigated. A total of 12 of the 2000 Paraguayan NBs were positive for one XAF1 p.E134* allele. Conclusions: The hypothesis of Spanish immigrants carrying p.R337H to Paraguay was disproved. TP53 p.R337H neonatal testing in Paraguay is not recommended, except when there are families with Brazilian ancestry presenting cancer cases. Additional epidemiological studies are required to determine the likelihood of the identified prevalence of the XAF1 p.E134* allele (1/153) in NBs from Paraguay without TP53 p.R337H to present cancer risk. This study complements the first national initiative for the DNA screening of newborns aimed at mapping the TP53 p.R337H and XAF1 p.E134* variants in Paraguay (based on the regions of residence of the newborns).

## 1. Introduction

Adrenal cortex carcinoma (ACC) is rare in children and linked to poor outcomes [1]. It often occurs in families with Li–Fraumeni syndrome [2,3], which involves various cancers due to TP53 gene mutations [4,5]. The specific genetic change in children from Paraná State (Brazil) is the p.R337H variant, which is inherited from parents [6]. In adults, this variant is less common and usually occurs in only 13% of cases [7]. The State of Paraná (Brazil) has the highest incidence of ACC in children globally, which is also significantly higher than that in other regions [8,9]. Environmental factors such as certain pesticides may influence the risk of developing ACC in children [10]. The p.R337H variant is widespread in Paraná, with over 80% of cases linked to it in São Paulo [11]. Our prior research in Paraguayan cities on the border with Paraná showed an incidence rate of 0.05% of the variant [12]. Surgical treatment is the main option for ACC, but chemotherapy is less effective in p53 mutations [13]. Complete tumor removal is crucial to better survival rates, especially in early-stage cases. The early detection of the tumor is challenging, making prevention strategies important [14].

The p53 protein was discovered in 1979 [15,16] and is linked to about 50% of human cancers [14]. It plays a key role in cancer by regulating cell cycle, apoptosis, and DNA repair. p53 helps prevent uncontrolled cell growth and maintains genome stability under stress [17,18].

Variants in the TP53 gene are found in many cancers, affecting its function [19]. TP53 variants can reduce p53 protein levels or change its activity [20]. Li–Fraumeni syndrome (LFS) is linked to inherited TP53 variants, increasing cancer risk, and is characterized by early-onset cancers, including sarcomas and breast cancer [4,21].

The high prevalence of p.R337H in southern Brazil is traced back to a common ancestor, highlighting genetic influences of cancer risk [22]. Evidence has confirmed that such a common ancestor is probably of Caucasian/Portuguese–Iberian origin, prior to the European migration to Brazil in the second half of the 19th century [23].

XAF1 has been identified as a new target gene of p53, which regulates its expression in normal cells but not in mutated ones. High levels of XAF1 can activate wild-type p53, leading to increased cell death and improved cancer response [24]. The loss of XAF1 expression is associated with advanced cancer stages, highlighting its role in tumor progression [25]. Recent research suggests that the co-segregation of the variant XAF1-E134* and TP53 p.R337H mutations leads to a more aggressive cancer phenotype than TP53 p.R337H alone [26]. Breast cancer may be mildly influenced by co-segregation with XAF1 p.E134*, and this variant can also confer risk for sarcoma [27].

ACC in children, especially in those under 4 years, has different causes and effects compared with adults, with 90% of cases showing hormonal symptoms, mainly isolated virilization [14]. A study showed that most cases in children are diagnosed between the ages of 2 and 4, while adults often have non-functioning tumors and conditions such as Cushing’s syndrome [14]. The p.R337H variant affects children more, leading to ACC, but is less harmful in adults, who usually develop ACC from other genetic changes later in life. Adult ACC is linked to aging and changes in adrenal glands, while children’s ACC is often due to early developmental issues [7,28].

**Figure 1:**
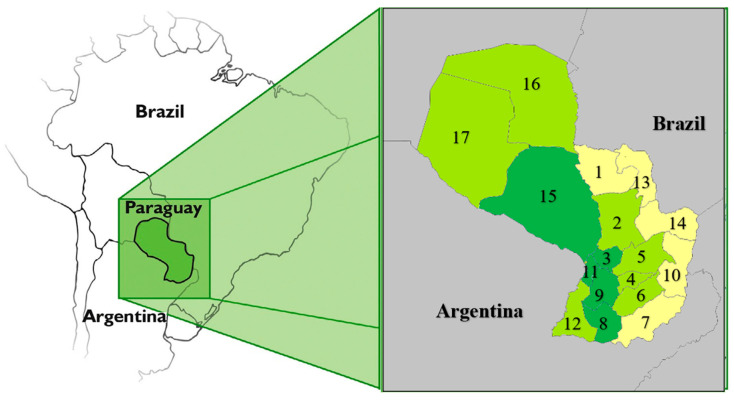
Paraguay area description for TP53 p.R337H variant and XAF1 p.E134* variant screening ^1^. ^1^ Paraguay (area inside rectangle) is subdivided into 17 departments (provinces or states). Blood samples from newborns screened for TP53 p.R337H variant were collected from towns located in Departments 2, 3, 4, 5, 6, 8, 9, 11, 12, 15, 16, and 17 (colored in dark and light green). Blood samples from newborns screened for XAF1 p.E134* were collected from towns located in Departments 3, 8, 9, 11, and 15 (colored in dark green). No blood samples were collected from towns located in Departments 1, 7, 10, 13, and 14 (colored in yellow) at the border with Brazil, which received most Brazilian immigrants in the past. This area was previously screened for TP53 p.R337H variant in [12].

Paraguay has a population of 6,109,903 [29], with an equal distribution of gender (3,057,674 men and 3,052,229 women). Most Paraguayans (95%) are considered mixed-race. There are no official statistics on ethnic composition, but studies show that Paraguayans are genetically closer to Spaniards than to native populations [30]. Paraguay is a landlocked country (Figure 1) of 406,752 km^2^, bordered by Bolivia, Argentina, and Brazil, and is divided into 17 departments, plus the capital (Table 1).

It was previously found that in the south and part of the southeast of Brazil, ACC prevalence is higher than in any other country. A notable migration of southern Brazilians to the Paraguayan region at the border of the Brazilian State of Paraná in the 1980s resulted in an estimated number of Brazilian descendants living in that region of about 300,000, and Brazilian official statistics counted 263,200 nationals living in Paraguay in 2023 [31]. A prior work of this research group in 2015 [12] showed high prevalence of the TP53 p.R337H variant in the Paraguayan cities on the border with Paraná, 0.05%. Thus, the question that remained at the time was what the prevalence of this variant was in the rest of Paraguay, which did not receive the same massive southern Brazilian immigration.

The main goals of this research study are to (1) estimate the prevalence of the germline TP53 p.R337H and XAF1 p.E134* variants in Paraguay (excluding regions on the border with Paraná State, Brazil, as this has already been researched) and (2) estimate whether the ethnic origin of TP53 p.R337H carriers in Paraguay is similar to that of ethnic groups in Paraná (possible Portuguese/Spanish origin).

## 2. Materials and Methods

Tests of the TP53 p.R337H variant in Paraguay: The research population comprised newborns (NBs) randomly selected from hospitals and maternity wards in all departments of Paraguay, including the capital, Asunción, located away from the border with Paraná from 2016 to 2019 (Figure 1). The analyses were conducted from February 2016 to October 2019, aiming to detect the p.R337H variant in TP53. This variant is closely related to the development of ACC in children, and given the experience gained in the border region between Brazil and Paraguay, where Brazilians and descendants of Brazilians reside, the choice was made to include the entire country’s newborn population for a comparison of the findings, excluding the regions of the prior experiment.

The sample type used for testing the TP53 p.R337H and XAF1 p.E134* variants was peripheral blood collected from the heel of newborns through the “National Neonatal Detection Program” of the Paraguayan Ministry of Health. The blood was stored on a special membrane (Whatman Protein Saver Card #903) (Cytiva, Marlborough, MA, USA), which was kept at room temperature and sent to Health Center No. 9 in Asunción (via Paraguayan postal service) for analysis and subsequent storage. The population selected for testing the XAF1 p.E134* variant comprised 2000 newborns from hospitals and maternity wards in five departments in regions far from the Brazilian border (Figure 1), located in the most populated areas of the country. The prevalence of the variant found was compared with prevalence rates recorded in Brazil and abroad. The XAF1 p.E134* variant corresponds to the SNP rs146752602 (chr17: 6,663,899, GRCh37/hg19), which is a stop codon gain variant (E134*/Glu134Ter), substituting G for T in the tumor suppressor gene XAF1, referred to as XAF1 p.E134*. This variant was searched in an open-access genomic database of University of São Paulo, which is based on the sampling of 1117 elderly individuals from southeastern Brazil who had never been ill or had cancer [32]. The prevalence of the germline variant XAF1 p.E134* in Paraguay was also compared with a database from NCBI—National Center for Biotechnology Information [33]—which includes samples from countries in Latin America and Europe.

The detection of the TP53 p.R337H variant was carried out by using a PCR-RFLP assay, as described in references [34,35]. In summary, 3 mm circular punches of filter paper containing dried blood from heel pricks were processed in duplicate within 96-well plates, with each well holding blood from two newborns. A 447 bp DNA fragment covering TP53 exon 10 was amplified by using PCR. The resulting amplicons were incubated in duplicate with HhaI endonuclease and then separated with electrophoresis. The TP53 R337H allele produced a single 447 bp fragment, while the wild-type allele generated two fragments measuring 154 and 293 bp. Exon 10 was sequenced in all neonatal samples that tested positive for R337H to verify the results. The tests were repeated if the gel visualization yielded ambiguous results, with common reasons for repetition including insufficient washing, reagent problems, or poor amplification due to sample interference.

A real-time PCR analysis for the XAF1 p.E134* variant was performed on the peripheral blood samples stored on filter paper collected from five departments across Paraguay. For the analysis, we utilized real-time PCR TaqMan^®^ (Thermo Fisher Scientific, Waltham, MA, USA) hydrolysis probes and two fluorophores (FAM^®^ and VIC^®^) (Thermo Fisher Scientific, Waltham, MA, USA).

Each PCR plate included four controls: a negative control (H_2_O), a normal control for XAF1 p.E134*, a heterozygous mutant control, and a homozygous mutant control. Positive samples were reanalyzed for confirmation.

The amplification reaction followed a specific protocol using the following elements:

- A volume of 2 µL of DNA (5 ng/µL);
- A volume of 5 µL of TaqMan^®^ Genotyping Master Mix 2X (Applied Biosystems^®^, Thermo Fisher Scientific Baltics UAB, Vilnius, Lithuania);
- A volume of 0.125 µL of VIC^®^/FAM^®^ probe (rs146752602) (Thermo Fisher Scientific, Waltham, MA, USA)
- A volume of 2.875 µL of ultra-pure water.

The PCR was conducted with an Applied Biosystems^®^ 7500 Fast device (Thermo Fisher Scientific, Waltham, MA, USA).

The presence of the mutant allele XAF1 p.E134* is indicated by the amplification of the FAM^®^ probe (blue signal). The normal allele is identified with the VIC^®^ probe (green signal). For homozygous variants, only the FAM^®^ probe can be amplified, while both probes can be amplified in heterozygous cases. In negative samples, only the VIC^®^ probe is amplified.

## 3. Results

### Results of TP53 p.R337H and XAF1 p.E134* Variants in Paraguay

▪ Prevalence of TP53 p.R337H

The analysis of the 40,000 samples collected from February 2016 to March 2019 revealed that there were no carriers of the TP53 p.R337H variant in Paraguay.

▪ Prevalence of XAF1 p.E134*

Out of the 2000 analyzed samples, 12 Paraguayan newborns were identified to have the XAF1 p.E134* variant, resulting in a prevalence rate of 6 per 1000 (0.6%). The distribution of cases by department is detailed in Table 2. Only four families of the identified cases could be contacted or accepted to be contacted, with varying health backgrounds among the children and their parents (Table 2).

▪ Prevalence of XAF1 p.E134* in Paraguay and Other Continents

In the absence of genomic databases for healthy Paraguayans, the prevalence of XAF1 p.E134* was explored in Brazilian databases [32], showing similar rates to Paraguay.

NCBI database insights: The XAF1 p.E134* variant, characterized by a single-nucleotide polymorphism (SNP), has a prevalence rate ranging from 0 in certain Latin American populations to 0.5% in Europe, illustrating significant geographical variability [33].

This comprehensive analysis highlights the absence of TP53 p.R337H in Paraguay and presents critical findings on the prevalence of the XAF1 p.E134* variant, contributing to the understanding of genetic risk factors in the region.

## 4. Discussion

Li–Fraumeni syndrome (LFS) is characterized by a strong familial history of cancer, including at least one case of sarcoma, as described by Li and Fraumeni in 1969 [36]. Individuals inheriting mutations in the DNA-binding domain of the TP53 gene (e.g., R175H or R248W) are at high risk of developing various tumors, such as breast, brain, soft tissue, and bone cancers, often at a young age [37].

The loss of the second normal allele of the TP53 gene may lead to the loss of tumor suppression in adrenal cortical cells [38]. In vitro studies have shown that the p53 protein with the p.R337H variant generally functions normally [6,39]. Research involving genetically modified mice expressing the equivalent variant (R334H) indicated low cancer risk, with these mice exhibiting elevated levels of the partially compromised p53-R334H protein in response to DNA stress. However, these homozygous mutant mice only displayed a slight increase in spontaneous tumor susceptibility, which was not statistically different from that of wild-type mice [37,40].

Li–Fraumeni-like syndrome (LFLS) is observed in approximately 30% of individuals with the p.R337H variant, often without sarcomas or meeting the age criterion of 45 years, as per [41]. The classification of less pathogenic TP53 variants, such as p.R337H, is complex, prompting the need to quantify the “spectrum of LFS” [42]. This approach accounts for the phenotypic variability of cancer within families, particularly regarding the p.R337H variant, which is frequently found in conjunction with the XAF1 p.E134* variant in about 70% of cases in Paraná, complicating the understanding of cancer risk [26].

The p.R337H variant is attributed to a founder effect likely originating from the Portuguese Jewish population, with high prevalence in southern and southeastern Brazil, and is also found in Portuguese and Spanish families [27]. Related research has revealed that this variant is older than previously thought and exists in multiple countries, albeit due to different founder effects.

Findings on TP53 p.R337H and XAF1 p.E134* variants in Paraguay: The initial hypothesis was that the p.R337H variant found in newborns from Paraguayan cities bordering Brazilian State of Paraná might indicate the presence of additional carriers with Spanish ancestry inside the country. This led to a study involving the screening of 40,000 newborns far from this border area, thus excluding Brazilian and mixed-ancestry newborns. All tests employed positive and negative controls, yielding strong amplification signals (447 bp) in dried blood samples. However, the p.R337H variant was not detected in this population, primarily composed of mixed-race individuals with Spanish descent geographically more distant from southern Brazil, disproving the hypothesis of its emergence from the Spanish side in Paraguay. This result is consistent with the conclusion in [28] that the frequency of R337H carriers is inversely proportional to the distance from Paraná and São Paulo States (located in southern Brazil). As stated before, the few positive cases found in Paraguay in our prior research work [12] were limited to cities bordering Brazil, with families having migrated from southern and southeastern areas of this country.

Testing for the XAF1 p.E134* variant was also conducted on Paraguayan newborn samples. The prevalence of p.E134* was found to be 0.6% (1 in 166), similar to rates in Brazil [32] and some European countries [33]. p.E134* may be pathogenic for sarcomas when it co-segregates with p.R337H on chromosome 17p13.1 [26].

This study underscores the importance of understanding the genetic landscape and cancer risks associated with these variants in distinct populations.

Future works should focus on the following: 1. research on the prevalence of the TP53 p.R337H variant in ACC samples from children from Paraguay, as well as pathologies related to the variant; 2. further research on the prevalence of the XAF1 p.E134* variant in other departments of Paraguay and the determination of its connection to the types of cancer observed; 3. investigations into the role of environmental factors in the origin of the p.R337H and XAF1 p.E134* variants (even though no de novo variant has been identified to date) and/or in the formation of ACC and other forms of cancer.

## 5. Conclusions

In analogy to what was performed in the State of Paraná (Brazil), this study complements the first national initiative for the DNA screening of newborns aimed at mapping the TP53 p.R337H and XAF1 p.E134* variants in Paraguay (based on the regions of residence of the newborns). This study was important to validate that the arrival of carriers of the p.R337H variant in Paraguay (linked to Paraná, where it has the highest prevalence) likely did not originate among Spaniards.

ACC is a rare tumor, but the state of Paraná (Brazil) has the highest incidence of ACC in children globally. This incidence is associated with the high prevalence of the TP53 p.R337H variant in Paraná (0.30%), which diminishes in the Paraguayan cities on the border with Paraná to 0.05%, without findings of the variant in areas of Paraguay more distant from this border. This result is consistent with prior research studies showing that the frequency of R337H carriers is inversely proportional to the distance from Paraná and São Paulo States in Brazil. This finding supports the conclusion that in areas of Paraguay far from the border with Brazil that did not have high immigration rates of Brazilians, ACC in children is as rare as in the rest of the world.

Carriers of the XAF1 p.E134* variant were identified in Paraguay among individuals without the p.R337H variant. The prevalence of XAF1 p.E134* in Brazilian databases [32] is similar to that in Paraguay. The relationship of this variant with cancer development risk should be assessed in new studies in the Paraguayan population, since it was reported to lead to more aggressive cancer phenotypes.

## Figures and Tables

**Table 1:** Paraguay population lineage and Brazilian immigrants ^1^.

Populational Feature	Geographical Distribution
Mixed race	Located in all departments except in central Chaco, where the majority of the population are Mennonites who have migrated mainly from Canada
Guarani and non-Guarani Indians	Mainly located in Departments 15, 17, 13, 14, 5, 4, and 6, in decreasing order
Brazilian descendants	Mainly located in Departments 10, 14, 7, 13, and 1
High-density areas	Departments and codes: Central (11) + Asunción = Capital (code ASU) + Alto Paraná (10) + Caaguazú (5), and Itapúa (7), in decreasing order
Low-density areas	Departments and codes: San Pedro (2) + Cordillera (3) + Guairá (4) + Concepción (1) + Paraguarí (9) + Amambay (13) + Misiones (8) + Canindeyú (14) + Caazapá (6) + Presidente Hayes (15) + Ñeembucú (12) + Boquerón (17) + Alto Paraguay (16), in decreasing order

^1^ Adapted from [12].

**Table 2:** Results of PCR-RFLP screen for TP53 p.R337H and XAF1 p.E134* variants in Paraguayan newborns ^1^.

Department	NBs Screened for R337H Variant	NBs Screened for XAF1 p.E134* Variant	XAF1 p.E134*-Positive NB
(2) San Pedro	3440	0	
(3) Cordillera	2442	173	4 ^(1)^
(4) Guairá	1833	0	
(5) Caaguazú	4510	0	
(6) Caazapá	1503	0	
(8) Misiones	1024	73	0
(9) Paraguarí	2163	153	0
(11) Central and Asuncion	20,727	1478	4 ^(2)^
(12) Ñeembucú	755	0	
(15) Presidente Hayes	963	69	4 ^(3)^
(16) Alto Paraguay	146	0	
(17) Boquerón	494	0	
Total	40,000	2000	12

^1^ TP53 p.R337H-positive NBs found: 0 for all 40,000 screened samples. Prevalence of XAF1 p.E134* variant was 0.6%. Sex of XAF1 p.E134*-positive NBs found: ^(1)^ four females; ^(2)^ two males and two females; ^(3)^ one male and three females.

## Data Availability

The data of the subjects are unavailable due to privacy or ethical restrictions.
